# Comment on Larroya et al. Validity and Reproducibility of a Spanish EPIC Food Frequency Questionnaire in Children and Adolescents. *Nutrients* 2024, *16*, 3809

**DOI:** 10.3390/nu18091422

**Published:** 2026-04-30

**Authors:** José María Huerta, María Dolores Chirlaque, Pilar Amiano, Marcela Guevara, María José Sánchez, Antonio Agudo

**Affiliations:** 1 Department of Epidemiology, Murcia Regional Health Council-IMIB, 30008 Murcia, Spain; 2 CIBER Epidemiología y Salud Pública (CIBERESP), 28029 Madrid, Spain; 3 Department of Socio-Sanitary Sciences, University of Murcia, 30100 Murcia, Spain; 4 Public Health Division of Gipuzkoa, 20013 San Sebastián, Spain; 5 BioGipuzkoa Research Institute, 20014 San Sebastián, Spain; 6 Instituto de Salud Pública y Laboral de Navarra (ISPLN), 31003 Pamplona, Spain; 7 IdiSNA, Navarra Institute for Health Research, 31008 Pamplona, Spain; 8 Escuela Andaluza de Salud Pública (EASP), 18011 Granada, Spain; 9 Instituto de Investigación Biosanitaria ibs.GRANADA, 18012 Granada, Spain; 10 Department of Preventive Medicine and Public Health, University of Granada, 18012 Granada, Spain; 11 Unit of Nutrition and Cancer, Catalan Institute of Oncology-ICO, 08908 L’Hospitalet de Llobregat, Spain; 12 Nutrition and Cancer Group, Epidemiology, Public Health, Cancer Prevention and Palliative Care Program, Bellvitge Biomedical Research Institute-IDIBELL, 08908 L’Hospitalet de Llobregat, Spain

We are writing regarding the recently published article in *Nutrients* 2024, 16, 3809 (https://doi.org/10.3390/nu16223809) by Larroya et al., entitled “Validity and Reproducibility of a Spanish EPIC Food Frequency Questionnaire in Children and Adolescents” [1]. The study reports on the relative validation of an adult version of an FFQ against 9 days of 24 h dietary recalls (24HDR), as the gold standard, in Spanish adolescents. While we acknowledge the critical importance of validated dietary assessment tools for epidemiological research on how dietary choices influence health, the paper contains a number of inaccuracies that we, on behalf of the EPIC-Spain group, feel compelled to clarify.

The European Prospective Investigation into Cancer and Nutrition (EPIC) Study is an ongoing prospective collaborative effort across 10 European countries (https://epic.iarc.fr/, accessed on 27 April 2026) with the main aim of providing a strong scientific basis for associations between diet and health outcomes, further providing insights into potential mechanisms of action. The study enrolled over half a million adult participants (aged 40–70 years) across Europe who provided comprehensive data on their lifestyles and clinical and reproductive history, in addition to a thorough evaluation of their usual diet. From then on, the incidence of cancer and other chronic diseases, and the vital status of participants, was periodically assessed to keep an updated prospective record of health events. The EPIC study represented a milestone for the study of the nutritional epidemiology of cancer as it was one of the largest epidemiological studies ever conducted, which remains at the forefront of epidemiological research.

As a study focused on nutrition and chronic disease, the assessment of the habitual diet of the participants was considered one of the most relevant dimensions of the baseline assessment. To this end, each country/center was allowed to design the dietary tool best fitted to their populations in order to gather data that were both comprehensive and representative of the habitual diet of the participants. Quantitative and semi-quantitative dietary questionnaires were used, including FFQ, 7- or 14-day records, and quantitative diet questionnaires structured according to meal occasions, often termed as the diet history method [2]. Therefore, strictly speaking, there is *not* an EPIC FFQ but rather country-specific diet questionnaires, mostly (but not exclusively) FFQs. These assessment tools were further validated in their respective populations and, later, they were calibrated by means of 24HDRs [3]. The previous two references provide a detailed description of the EPIC study and the calibration study, respectively [2,3].

In their *Nutrients* paper [1], the authors state that they used a ‘Spanish EPIC-FFQ’, which is inaccurate or, at least, confusing. The Spanish centers (Asturias, Granada, Murcia, Navarra, and Gipuzkoa) never used an FFQ for dietary assessment, but a comprehensive quantitative Diet History [4], which has been extensively validated [5,6,7], and shown to perform better than other self-administered FFQs in capturing habitual dietary intake and its association with disease outcomes. The authors seem to have mistakenly taken the FFQ designed within the EPIC-Norfolk cohort and assumed the same questionnaire was used in Spain. Furthermore, the authors cite three articles (references 15, 20, 21 in the paper by Laroya et al., 2024) in support of the validation of this FFQ in adult Spanish participants, but these references have also been misused: two of them validated the respective EPIC FFQs applied to UK or Italian populations, while the other (Kaaks et al., 1997, reference 15) actually deals with the methodological aspects of calibration and validation in dietary intake measurements. As mentioned, neither of the EPIC-Spain cohorts ever used an FFQ for dietary assessment, nor has the EPIC-Norfolk FFQ ever been validated for use with adult Spanish population. A more precise statement would be as follows: “While different EPIC questionnaires have been validated for use in European adult and adolescent populations, the reliability and validity of the EPIC-Norfolk FFQ for Spanish children and adolescents has not been established.”

The study by Larroya et al. effectively shows the relative validity of a Spanish version of the EPIC-Norfolk FFQ to assess the dietary intake of Spanish children and adolescents; this letter is not intended to question the methodology or the results presented. Even when several valid FFQs have been developed and validated in young Spanish population that they could have opted for [8,9], we value the work of the authors as a relevant contribution to the field. However, and in order to prevent any confusion regarding the nature of the ‘Spanish EPIC-FFQ’, we believe that it is important to clarify that the FFQ used in their publication was designed within the EPIC-Norfolk study in the United Kingdom, not EPIC-Spain, and that this FFQ has not been validated for use in adult Spanish populations (a claim that the authors and the publisher are encouraged to amend via an erratum to the paper). We believe that the wording of the title and the manuscript is confusing regarding this point and may mislead readers regarding the actual dietary methods used in the EPIC-Spain cohort or the existence of a Spanish EPIC-FFQ. We would kindly suggest that the authors make an express mention of the EPIC-Norfolk FFQ in the title and ‘objectives’ paragraph. By agreeing to these corrections, the authors would avoid any misunderstandings arising from their otherwise meritorious paper.

Scientific dialog is essential for advancing knowledge. On behalf of EPIC-Spain, we wrote this letter to clarify to potential readers of Larroya’s work that there is no such thing as a Spanish EPIC FFQ. Therefore, referring to a “Spanish EPIC FFQ” could mislead readers to assume that such a standardized instrument exists across all EPIC centers, which is not the case. We would be very much grateful if the authors would consider the suggested changes positively and take the necessary steps to avoid future misunderstandings regarding the existence of this dietary instrument, and we are more than happy to collaborate in achieving this goal. The aim of this letter is not to criticize the quality of the work conducted by the authors. They appropriately adapted and used a Spanish version of the EPIC-Norfolk FFQ, which is an acceptable and adequate approach. Furthermore, they demonstrated the relative validity and reproducibility of the adapted instrument in the Spanish youth population, thus providing the scientific community with a useful tool. Explicitly stating the instrument used in the title would only improve scientific precision and avoid any potential misunderstandings. Finally, we believe that the original EPIC-Norfolk questionnaire, the Spanish version of which is the one validated in the article, should be duly accredited. There are numerous references regarding different aspects of this instrument, the most comprehensive one perhaps being Bingham et al., 2001 [10].
